# WSN- and IOT-Based Smart Homes and Their Extension to Smart Buildings

**DOI:** 10.3390/s150510350

**Published:** 2015-05-04

**Authors:** Hemant Ghayvat, Subhas Mukhopadhyay, Xiang Gui, Nagender Suryadevara

**Affiliations:** SEAT, Massey University, Palmerston North 4442, New Zealand; E-Mails: ghayvat@gmail.com (H.G.); X.Gui@massey.ac.nz (X.G.); suryadevara99@gmail.com (N.S.)

**Keywords:** IOTs, wellness function, behavioral detection, attenuation loss, interference, SNR (signal to noise ratio)

## Abstract

Our research approach is to design and develop reliable, efficient, flexible, economical, real-time and realistic wellness sensor networks for smart home systems. The heterogeneous sensor and actuator nodes based on wireless networking technologies are deployed into the home environment. These nodes generate real-time data related to the object usage and movement inside the home, to forecast the wellness of an individual. Here, wellness stands for how efficiently someone stays fit in the home environment and performs his or her daily routine in order to live a long and healthy life. We initiate the research with the development of the smart home approach and implement it in different home conditions (different houses) to monitor the activity of an inhabitant for wellness detection. Additionally, our research extends the smart home system to smart buildings and models the design issues related to the smart building environment; these design issues are linked with system performance and reliability. This research paper also discusses and illustrates the possible mitigation to handle the ISM band interference and attenuation losses without compromising optimum system performance.

## 1. Introduction

Innovations in technology mostly emerge from the needs of human society. The 21st century is the era of prompt advancement in digital technology. Most of this technology is focused on proficiently monitoring and controlling different activities. Everywhere from mega-structure building automation to small smart homes, big industrial assembly machineries to a kid’s toy, a college research laboratory to an international space research center, and even health care service at a desk through wireless sensors and networks, wireless sensor networks (WSN) have become fundamental and crucial devices. The significant improvement offered by introducing wireless technology is that it reduces the complexity to harness wired transmission and facilitates the installation of sensors, controllers, and actuators. The cost and installation efforts for a large number of sensors in an urban environment are exponentially reduced by wireless technology innovations. There are different wireless communication mediums (technology) in which a wireless sensor network can be constructed according to respective applications and strengths.

Home automation and monitoring are the dominant applications of WSNs, where a number of heterogeneous sensors are deployed, to determine different activities of inhabitants. Wireless sensors can be operated through batteries as well as plugging into the power supply. The selection of power source depends on the deployment environment and the availability of power for applications such as solar panel based outdoor temperature sensing. In a replaceable battery, the sending power and sampling rate of data decide battery life while the energy harvesting applications use the external energy source, so they are somewhat free from the battery usage method. Wireless sensors and networks are comprised of several nodes prepared through various sensing devices (sensors, controllers, and actuators) and RF chips for wireless communication [1,2,3,4]. A revolutionary development to WSNs is the announcement of IEEE 802.15.4 standard in the year 2003 [5], and it is the first major worldwide standard for WSNs. The limitation of IEEE 802.15.4 standard is that, it only specifies and defines the RF communication with respect to the lower layers PHY and MAC; it provides proposals and does not define the networking techniques for the upper layers. In light of this, ZigBee Alliance and its Mesh network along with IPv6 (in recent years) standardized the protocol known as ZigBee communication protocol (IEEE 802.15 ZigBee protocol). These upper layer improvements offer authentication of network nodes, encryption, and an efficient and modern routing that leads to mesh networking topology. Although mesh topology is quite complex, ZigBee is mostly preferred by WSNs designers with Mesh topology only. IEEE 802.15.4 ZigBee offers tremendous specifications to short range and urban environment wireless sensors networks. IEEE 802.15.4 ZigBee standards operate on the license-free industrial, scientific, medical (ISM) frequency band.

One of the significant developments in WSN-based smart homes is the injection of IOTs (the internet of things). The ubiquitous connectivity and distributed intelligence of the IOTs with wireless sensing technology are becoming the center point of upbeat remote monitoring and control. In an era of IOTs, more devices are linked to the internet as compared to the population—there were over 12.5 billion devices in 2010. Cisco predicts that 25 billion devices are going to come onboard in 2015 and there will be 50 billion by 2020. The internet of things from a smart home perspective can be described as connecting household objects, especially electronic and electrical appliances and sensors and actuators, to the World Wide Web services [6,7]. These objects and devices are smartly and intelligently interlinked to each other to develop new customs and understanding of wireless communication between a person and things, and among things themselves. These customs and understanding create a prospect for devices nearby in the smart home environment to start the interaction and build very different environments. An environment that learns from our daily activity and requirements, such as when we get up in the morning, how much time we sleep, and how long we watch television, can react to an inhabitant’s behaviour to improve their health and wealth. For example, an occupant got up late in the morning and because he or she was in hurry to catch the bus to the office, he/she forgot to turn off the electric oven and only noticed this after reaching the office. Now from a remote distance, through an IOT-based smart home system, he or she can control the oven. 

The motivations to design and develop the smart home are: independent living; enhancing comfort, efficient use of electricity, and safety and security. The word ‘smart home’ is preferred for a home environment equipped with advanced technology that allows monitoring and control of its inhabitants, and boosts independent living through wellness forecasting based on behavioral pattern generation and detection. To identify the difficulties and challenges of the prime performance of smart home monitoring, we have to understand recent and ongoing research in this field. A variety of smart home systems for ambient assisted living has been proposed and developed, but there are, in fact, relatively few houses that apply smart technologies. One of the main reasons for this is that the complexity and varied design requirements associated with different domains of homes. These domains are communications, control, entertainment, residential and living spaces. AHRI (Aware Home Research Initiative) at Georgia Institute of Technology [8], CASAS (The Center for Advanced Studies in Adaptive Systems) at Washington State University [9], AgingMo at University of Missouri [10], PlaceLab at MIT [11] and Smart home Lab at Iowa State University [12,13], are monitoring the activity of daily living, enhancing the comfort, and creating context-aware relative scenarios through heterogeneous sensor deployment, and these sensor units include cameras as well. These are academic research projects. From a social point of view, the use of the camera is a direct violation of privacy for monitoring in a smart home environment. At the same time, the video stream by the camera is technically complex. Monitoring and analysis through video stream are simple, but it demands significant storage space on the server. The server designed at home is a local home gateway server, which has limited storage and collection memory. There are many different industrial big building smart home services available; Toyota’s dream house at Japan, e-House by McDonough, in New York, House R128 by Intgeniere, W.S at Stuttgart, Germany and Crystal House by Hung, at Taichung, Taiwan, which have targeted sustainability and energy conservation goals, and offer convenience and comfort through the intelligence of the environment [14].

Wearable, implantable, and microsystems that can be deployed over the body area network such as the Apple watch are available nowadays. These devices are worn by the individual or embedded in the home environment to assist someone for health care. Several projects comprising the application of signal assisted wearable devices are ongoing in different research laboratories, SmartShirt [15], Senswear Armband by Body Media Inc [16] and LifeShirt by Vivometrics [17], PROETEX project by CSEM center [18] are wearable devices to measure ECG. Other research has focused on fall detection based on accelerometers using wearable sensors [19,20,21,22,23,24]. An individual suffering from illness can wear these devices as part of a health care strategy, but someone who is fit may not appreciate wearing these devices on their body. Adaptability is a significant concern with wearable sensor devices for physiological parameter measurement. 

Other researchers have already implemented smart home systems based on wireless communication technology, but integrating wireless with IOTs has extraordinary potential in smart home applications. Most research of IOT-based smart homes are in the proposal phase, and very few research studies involve practical implementation [25,26,27,28,29,30,31]. IOT-based smart systems offer us remote access and reactive ambient analysis of home ambience, but they can only perform this when the implementation of IOTs is done through dynamic database management [32,33,34,35]. Additionally, the majority of smart home projects are carried out systematically based on the assumptions from laboratory or test bed controlled environments, which may be entirely different from a real environment where an inhabitant lives regular life regardless of sensor node installation. 

The smart home environment demands activity recognition of daily living from raw sensor data, and these raw data sets are complex and irregular to encode into predefined scenarios. Even after encoding this raw data, it is quite difficult to identify deviant behavior because these data sets are on different time measures (sampling rate) and sense modalities. These different time and sensor modalities create problems during equation formulation. It is quite easy to generate behavioral patterns from single event data sets, but it may raise a false alert. In our research, we present a realistic approach to getting the optimal performance from a smart home monitoring system. Realistic application for behavioral pattern generation for ambient-assisted living raises different challenges. To provide a reliable solution, we proposed and implemented a smart home based on an integrated framework to analyze the individual’s behavior from the previous data, real-time recently received data and feedback received data. To resolve the issues, we apply pattern detection through pipeline processing with filtering, characteristic construction, activity detection, and smoothing. We attempt to separate the usual routine data from unexpected data, which may put an inhabitant’s health and wealth at risk. 

The system is proposed and implemented on two levels; hardware and software. At the hardware level, heterogeneous sensors are deployed for multi-activity and multi-events; these wireless sensor nodes are configured with ZigBee Mesh topology and data is received by central coordinator node and collected by local home gateway computer. The software modules are subdivided into different levels, such as data logging, data extraction, and data storage; but their ultimate task is to forecast the change in activity and correlate it with the wellness of inhabitants in real time or near time. 

In the second half of the paper, we have extended the smart home approach to big building environments and investigated performance and issues, especially interference and attenuation issues. Smart home monitoring is based on the ambient scenario; when extend our research to smart buildings we are faced with some new problems that we do not notice or consider in a smart home. 

We can define the intelligent and smart monitoring as an application of automation with integral systems of accommodation facilities to boost and progress the everyday life of an occupant. In this research, we have designed an XBee series-2 based intelligent monitoring system that operates on the ZigBee protocol and uses the features of IOTs. Our idea is to generate a system-level design approach to formulating design issues and possible solutions. The remaining parts of this paper are organized as follows. We start by presenting the existing system in Section 2 and experimental results and performance issues in Section 3. The extensions of a smart home for big building environments are presented in Section 4. Measurements and investigations from big building environment experiments are presented in Section 5. Lastly, a conclusion with possible solutions to issues is drawn in Section 6.

## 2. Description of the Developed Smart Home Monitoring System 

Smart homes for ambient-assisted living involve the learning patterns of agent behavior from sensor data. Deviation from the regular behavior is an array of the data that either does not follow the anticipated behavior, which is an anomalous behavior, or follows the certain class of predefined irregular behavior. This deviation is an alert for an inhabitant. In our research for behavioral pattern generation, we used an advanced approach that is based on combined deviation over time because forecasting can be reliable only if we consider more than one event. The wellness forecasting model we have designed and proposed is divided into two sections. At the lower section, all the raw sensor data from household appliance usage and movement is delivered to the coordinator attached to the local home gateway and this local server stores the unstructured data sets for further processing and analysis. Raw sensor data collected at the lower information section can only identify which sensor is active or inactive and at what time. To discover the optimal activity of daily living for wellness, these data sets need to be analyzed by the upper section software logic. Intelligent data recognition and classification mechanisms are applied through the software at different levels of data generalization in real time, based on the time and order of the sensor usage.

There are various ZigBee RF modules available in the wireless industry. For our research application, we pick Digi XBee Series-2 RF. The XBee module facilitates various features such as different sampling rate, baud rate and sleep rate associated with two types of operating modes. The first is an application programming interface (API), and the other is application transparent (AT).

### 2.1. Topology and Device Configuration 

Each ZigBee based wireless sensor networks has a ZigBee Coordinator (ZC). There is precisely one coordinator in every single network, and ZC is the device that takes the network establishment responsibility. ZC stores the network information including security keys (such as personal area network identification number). This coordinator is the transceiver RF module that not only receives the data from respective sensor nodes belonging to it, but also looks after remote configuration and fault detection of other associated sensor nodes in the network. In the tree and mesh topologies, the ZigBee routers (ZRs) is applied to extend the network coverage area for wireless communication. In addition to ZC and ZRs, there are two more types of ZigBee devices. The ZigBee device can be configured (programmed) as ZigBee end device (ZED) and ZigBee end device plus router (ZEDR). ZED is usually low power as well as small battery power devices. They transfer their data directly to the parent (ZC), and the parent may be the coordinator or another router node. The ZED cannot relay data from other nodes. The ZRs relay the packet of their neighbor to designated path while the ZEDR node can relay its data and other neighbors’ data as well in the network. The ZR and ZEDR nodes both have the same power option as ZED. The ZigBee nodes are scattered at different distances in the urban area of the home environment, and data is sent over the network to the coordinator.

### 2.2. Forming a Network

ZigBee based Digi XBee Series 2 is used as RF module in our smart and intelligent home monitoring system. The system contains heterogeneous sensors, which include PIR, temperature, force and electrical and electronic appliances usage monitoring sensors. The ZC has the authority to select a channel, PAN ID (16-bit personal area network unique identification number that only belongs to particular ZigBee WSN), security policy, and stack profile for the network. Each time to establish communication, ZC starts with the proper channel and executes a series of scans (energy scan or PAN scan) to realize any RF activity on various channels of ZED, ZR and ZEDR. This energy scan can only register the wireless sensor nodes (ZED, ZR & ZEDR ) which are registered with corresponding PAN ID to the ZC.

### 2.3. Channel Selection and Operation Mode 

It is the duty of ZC to select the best channel for network operation. To do this, it performs energy scans on every possible frequency channel (ZigBee 16 channels) to detect the strongest available channel. Channels at excessive energy levels are discarded from potential channel lists to start on. The type of operation mode depends on the individual’s requirements. It can be configured by the RF module through XCTU software [36]. 

### 2.4. Present System Description 

**Figure 1:**
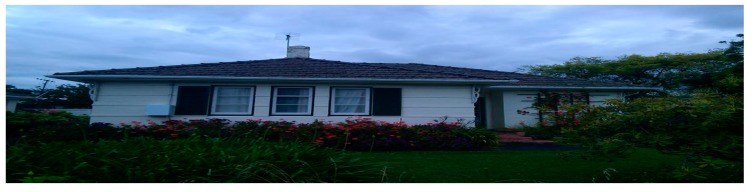
Image of real house where smart home monitoring and a control system are installed.

**Figure 2:**
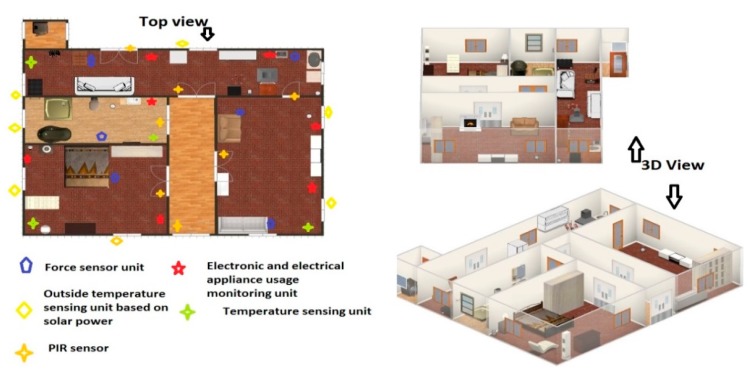
Layout structure of the house with sensor deployment and household objects.

The present home monitoring system has been continuously running since May 2013 without any significant complexity and maintenance, except minor problems related to sensor physical damage and power supply. Figure 1 represents the image of the real smart home where a monitoring system is running, and inhabitants are living their ordinary daily life. This house was built in 1938, so it is an old home, but it has been converted to a smart home with the help of sensing technology. The layout of the smart home with sensor location and placement is shown in Figure 2. This figure displays the genuine home environment where one stays alone and has smart sensing placement to improve wellness of individual, *i.e*., Ambient Assisted Living (AAL), and it shows different sensing units for monitoring of ambient parameters and different domestic objects. As we can see in the present system, it contains a network of heterogeneous sensors, These heterogeneous sensors are temperature, force, PIR and electronic and electrical (E & E appliances sensing) appliances monitoring units; Brief description of each sensing unit are given as follows:

#### 2.4.1. Electronics and Electrical Appliance Monitoring Unit 

**Figure 3:**
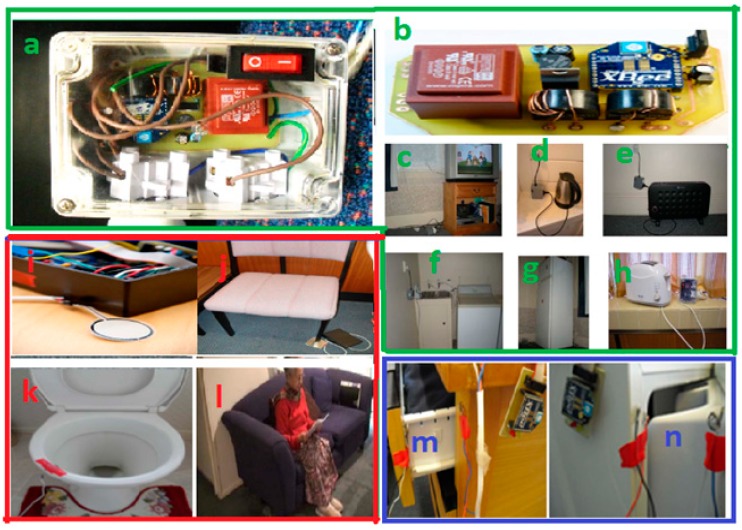
(**a**,**b**) represent the electronic and electrical appliance usage monitoring and control unit and (**c**–**h**) represent the monitoring and control of household appliances through this unit; (**i**) represents the force sensing unit and (**j**–**l**) deployment of the unit for different sitting as well as sleeping objects; (**m**,**n**) represent the contact sensing unit arrangement [39].

This power usage monitoring and control unit, contains transformer and other circuit components. The transformer block comprises of voltage and current transformers. The step down voltage transformer is used to transform the mains from 220 V to 10 V signal, and the current transformer ASM010 is used to link the current in the line wire to the load through the current transformer circuit. For signal amplification, operational amplifier LM324 is applied associated with other components such as a rectifier, capacitors and gain resistors of specific parameters. The analog sensor signal output is supplied to the analog channel of radio communication chip for wireless transmission. Figure 3a,b presents the designed unit, and Figure 3c–h shows the utilization of the unit with different household appliances. This data is helpful to analyze the appliance usage pattern [37]. 

#### 2.4.2. Force Sensing Unit

The flexiforce sensor is a Piezoresistive sensor A301 used to calculate the amount of pressure given on any object. When any pressure is given to the sensor, the resistance of the sensor decreases. As a result, the output voltage increases. The range of resistance varies in force sensor found in different types of sensors. The pressure is ideally given in the central circular part of the sensor on both sides. The sensor is kept beneath the objects to sleep and sit upon. The sensor is connected to a conditioning circuit with the 9 V power supply. Figure 3i shows the force sensing unit and (j) to (l) display the deployment of this unit to measure force. This force measurement is linked and analyzed to identify the occupancy pattern [38,39].

#### 2.4.3. Contact Sensing Unit for Domestic Objects 

For the purpose of domestic objects usage monitoring such as self-grooming table and office desk, we fabricated wireless contact sensing systems and connected them. Figure 3m,n shows the fabricated contact sensing unit connected to a grooming table to identify the frequency of usage, and these objects usage are monitored at local home gateway server by ON/OFF values [39]. 

#### 2.4.4. Temperature Monitoring Unit

**Figure 4:**
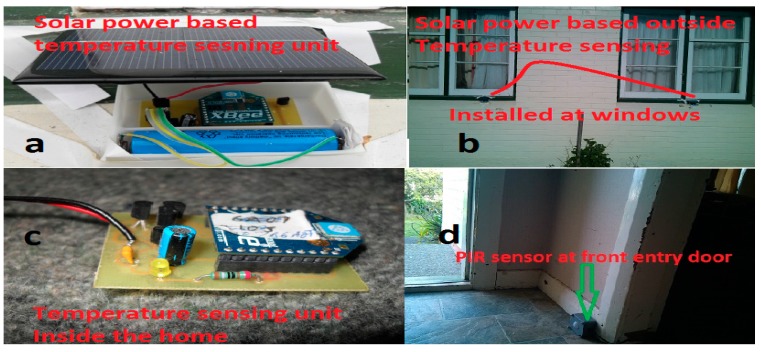
(**a**,**b**) represent the outside temperature monitoring unit based on renewable solar energy; (**c**) inside temperature monitoring unit and (**d**) PIR sensor for activity monitoring.

To design temperature sensing unit the LM35 IC is connected to conditioning circuit for ambient temperature monitoring. Figure 4a,b represents the outdoor temperature monitoring and Figure 4c shows the indoor room temperature monitoring. 

#### 2.4.5. Movement Monitoring Unit 

The passive infra-red (PIR) movement monitoring unit is designed to detect the motion within the coverage range of the sensing system. This PIR sensing unit is compact, power-efficient, flexible and durable. These sensing units are also known as “IR motion detector”. It operates on 5 V–12 V supply. These are binary mode sensors, and they are interfaced with RF XBee modules. Figure 4d shows the fabricated movement monitoring unit deployed in the smart home at the door.

**Figure 5:**
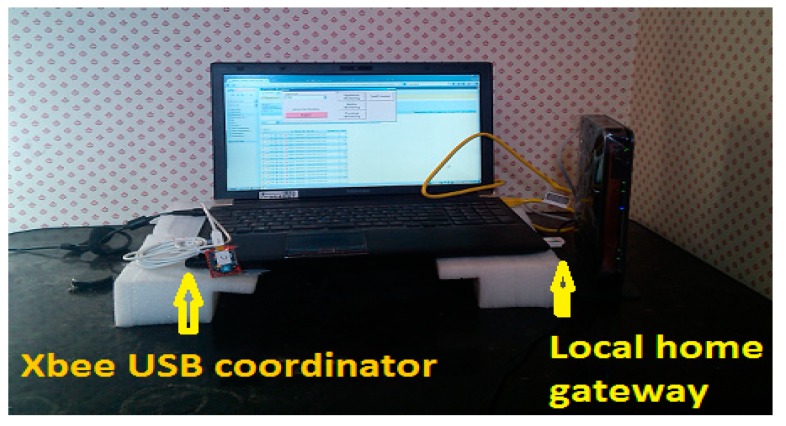
Local home gateway server computer.

The sensors are scattered in such a manner so that the systems get every essential value from every corner of the ambient environment that is applicable to wellness determination of an individual who stays there. Figure 3 and Figure 4 show the sensor units’ placement in the smart home. These sensing units transmit their data to RF module XBee series-2 end-device/router to XBee Coordinator connected (XBee coordinator connected to the laptop through USB connection) to home gateway server laptop. Figure 5 represents the local home gateway server laptop located in the smart home. The data from local MySQL server is raw data, from this raw data information is extracted through software. The software is designed according to the individual requirement of data extraction. The Visual Studio C# graphical user interface is designed to upload raw data to MySQL server. From the local server, that data will be analyzed and uploaded to the website with the help of the internet [40,41].

**Figure 6:**
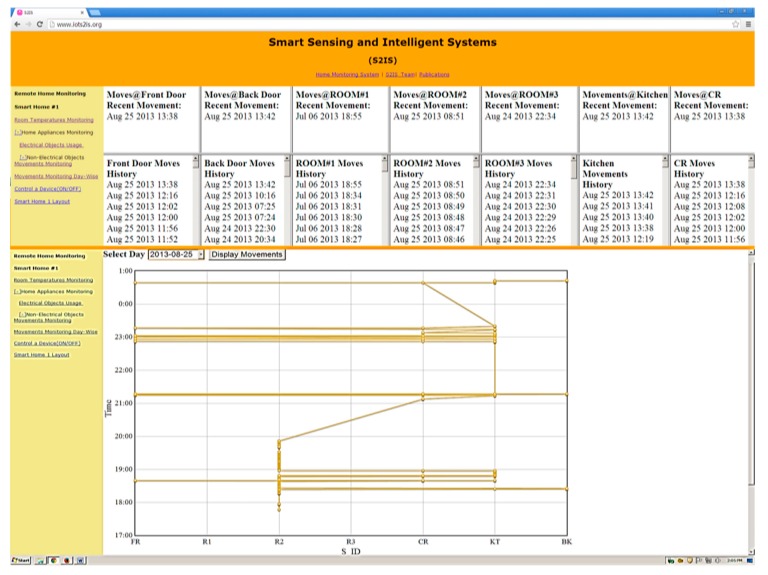
Online smart home monitoring website [39].

Figure 6 represents the movements of a subject on a particular day. The collected passive infrared motion sensor data files were processed and analyzed by sensor activity pattern generation software. The aim of this process was to track the movement of an elderly person and infer the top sequence of movement sensing unit at a particular time (a day or hour of the day). We had restricted the number of movement sensing units, and it was a beneficial approach to identifying the physical location of the person in real-time. We had placed the movement sensing unit exactly at the entry or exit door so that we could quickly identify the physical location of a subject.

### 2.5. Design of ADLs’ Recognition System

The ADLs’ recognition system design comprises the native element of the appropriate statistics of the ADLs. 

Sensor Event Level 0: This level covers a variety of sensing units deployed in the AAL environment. These are primarily used to produce vital data to the superior level. It transports the heterogeneous sensor data related to the subject statistics and directs the data to the contextual recognition level for classification.

Context Recognition Level 1: This level takes out the information from raw data and, depending on the AAL set-up’s principal values of location (S), time (T) and context (C), the contingent information is derived from the identification of Basic ADLs. 

ADLs Recognition Level 2: Classification for the Basic-ADLs will be achieved depending on the contextual information and the status of the sensor stream.

**Figure 7:**
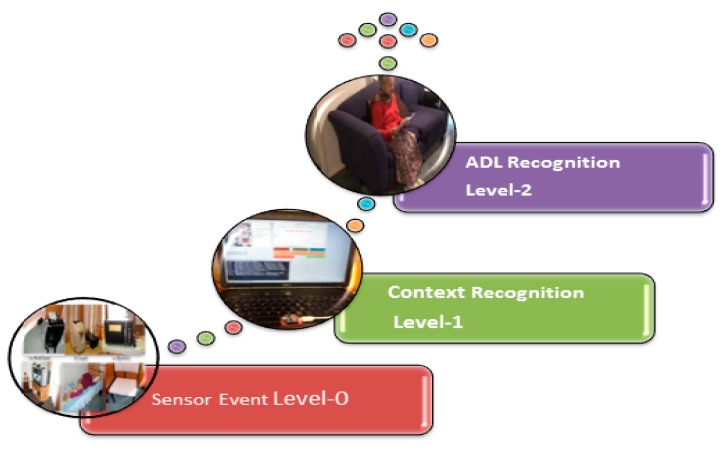
Activity recognition divided into three different levels [39].

At the lower section Level 0, all the raw sensor data from household appliance usage and movement is delivered to the coordinator attached to local home gateway and this local server stores the unstructured data sets for further processing and analysis. Raw sensor data collected at the lower information section can only identify which sensor is active or inactive and at what time. To discover the activity of daily living for wellness, these data sets need to be analyzed by the upper section software logic. The context (Level 1) is intended for recognizing the context at which the sensor value is generated. This level extracts the information from the sensor data and depending on the AAL set-up is the basic values of location, time and context, the situational information are derived from the identification of the respective ADL. The ADL (Level 2) is meant for recognizing the activity based on the context and sensor value, and the labeling for the basic ADL recognition will be performed at this level. Figure 7 shows the recognition of ADLs. It involves a Sensor Event Level, Context Recognition Level, and ADL Recognition Level [39].

### 2.6. Wellness Functions

Wellness functions are introduced to provide a mathematical expression and to ascertain the wellness of an individual under the ambient-assisted real-time monitoring environment. The two wellness functions β_1_ and β_2_ define the wellness of an inhabitant based on the usage of household appliances. The rationale for the wellness functions is to determine “how well” the inhabitant is using daily objects. The first function β_1_ is obtained from the non-usage as well as the inactive duration of the appliances. While the second function β_2_ is generated from the over-usage of few specific appliances. The wellness index shows the behavior of the person with respect to the daily object usages in real-time.

**Figure 8:**
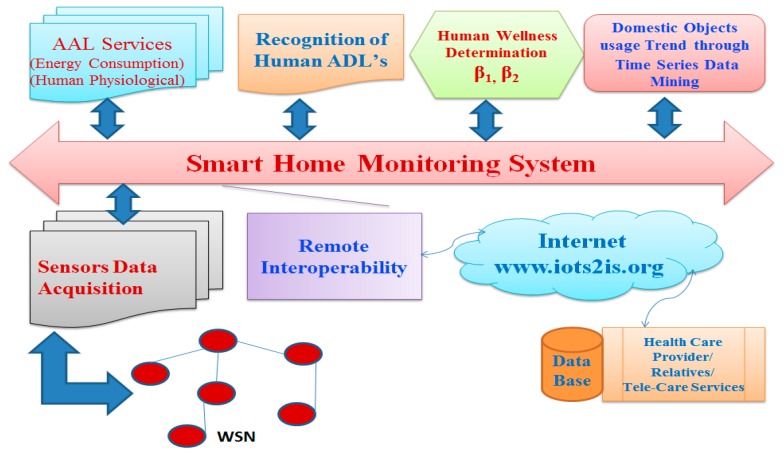
Functional description of the developed smart home monitoring system [39].

Figure 8 shows the functional description of the wellness-based smart home monitoring [37,38,39,40,41]. The smart home systems have been developed to observe the wellness of elderly people living independently in their own at home. The smart home system developed is capable of simultaneously monitoring the general physical activities of an inhabitant as well as physiological and ambient entities. It is a multi-model, unobtrusive, non-invasive novel sensing system deployed at central locations in the home environment. The continuous in-home monitoring can be achieved with single local home gateway server computer. The developed analysis and decision-making algorithm software modules execute in a Windows software working environment. Through the internet connection, we can access the wellness information from a remote location. 

Wellness function #1, designated as (β_1_) is defined by the following equation:

(1) β1=e−tT

where: ${\text{β}}_{1,\text{New}}$ = Wellness index of the elderly person based on the measurement of inactive duration of household objects; t = Time of Inactive duration of all appliances, *i.e*., duration time no objects are used; T = Maximum inactive duration when no objects were used in the past.

Wellness function #2, designated as (β_2_) is defined by the following equation

(2) $$\beta_{2}=e^{\frac{\left(T_{n-T_{a}}\right)}{T_{n}}}$$

where: β_2_, new = Wellness function of the elderly person based on excess usage measurement of household object; T_a_ = Actual (current) usage duration of the household object; T_n_= Maximum usage duration use of household object in normal situation of the past [39].

Seasonal Decomposition:

The seasonal (cyclic) decomposition by Brockwell and Davis in 2001 was developed for predicting the near future values. It has been applied as a primary tool to analyze trends. It is also useful for determining seasonal patterns, which improves the forecasting process. Trend component can assess using the principle of moving average. 

The initial Exponential Moving Average considered in the analysis is given by the equation below:

MA_t+1_ = αX_t_ + (1 − α) MA_t_ (3)

where:

MA_t+1_—Moving average prediction;
MA_t_—Previous Moving average;
α—Smoothing Constant;
X_t_—Observed quantity at time ‘t’.

The smoothing constant (‘α’), is derived from the number of sensor observations, and these observations contain the initial value from the start of the system to the recently observed value. The essential features of trend and seasonality are described in a time series by its degree. After assessing the internal components such as trend and seasonality of a time series, errors’ estimation is extracted by the de-trending process. Smoothed Trend Curve (STC) for various household usage durations is derived by applying Equation (3).

The seasonal decomposition is suitable for data revealing a cyclical pattern as well as a trend. In the present research task, a one-week activity duration series is considered as one cycle or season to recognize the weekly activity pattern of an individual. It has also categorized the periodic components in the historical data and used them in a forecasting model [39].

Driving Trend using Modified Double Exponential Smoothing Process: 

To smooth the trends, a modified double exponent smoothing strategy (Brockwell strategy) is applied for forecasting. The advantage of this strategy is to minimize the mean deviation and capture the local (latest seasonal) trend of the series.

(4) $$\tau =T=\delta \left(L_{t}-L_{t-1}\right)+(1-\delta )T_{t-1}$$

(5) $$L_{t}=\alpha \left(x_{t}-S_{t-s}\right)+(1-\alpha )(L_{t-1}+T_{t-1})$$

(6) $$S_{t}=\gamma \left(x_{t}-L_{t}\right)+(1-\gamma )S_{t-s}$$

where:

T_t_: trend (or slope) of the entire duration, L_t_: local level seasonal slope, S_t_: change in seasonal factor, x_t_ is the observation at the current time, s is the number of periods in one cycle (*i.e*., s = 7 in our case), α, δ, γ is the smoothing parameters ranging from 0 to 1, selected by minimizing mean square errors.

Starting values are: L_t_ = (1/s)(x_1_ + x_2_ + x_3_ +…..x_s_); T_t_ = (1/s)((x_s+1_ − x_1_)/s+ (x_s+2_ − x_2_)/s +…. (x_2s_ − x_s_)/s); S_t_= x_k_ − L_s_, where k = 1, 2….s.

A forecast of the activity duration is extrapolated by using the seasonal pattern in Equations (3)–(5):

F_t+m_ = L_t_ + T_tm_ + S_t__−s+m_ (7)

m is the required forecast period [39].

## 3. Experiment and Analysis of Smart Home 

The wellness trends and pattern generation for the activity of daily living are based on the real system running continuously in the real smart home since May 2013. The sensor data is collected into local home gateway computer and analyzed through the wellness function’s mathematical formula in Equations (1)–(7).

**Figure 9:**
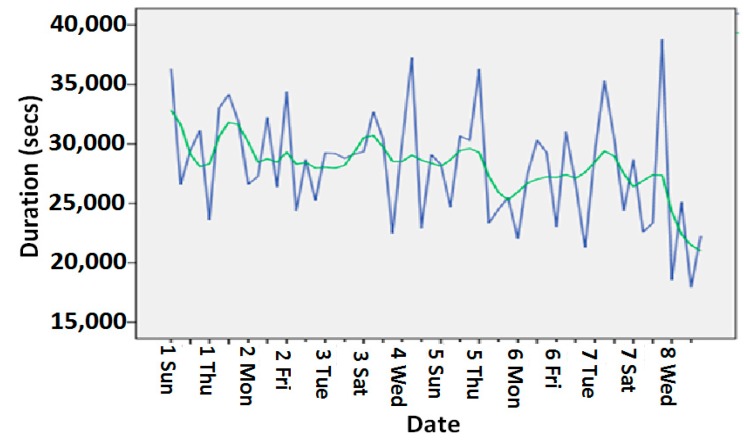
Bed usage durations and trend.

In the forecasting process, the most appropriate fitted curve is computed by adding smoothed trend curve and seasonally adjusted factors. For illustration, the non-electrical appliances usage durations and their corresponding trends are considered in the forecasting process. This would reveal the precise behavior of an individual in utilizing the household appliances. Some of the electrical appliances such as water kettle, microwave, and laundry machines are pre-programmed and auto control; hence, they may not appropriately run the forecasting method. The non-electrical appliances usage duration and their corresponding trends are plotted. Figure 9 shows bed usage activity durations and its corresponding trend for eight weeks at the smart home where an inhabitant lives their regular life (green color: trend; blue color: actual observations) [37,38,39,40,41,42]. The trend we generated from our wellness model fitted to the actual observations. With these trends, we can predict the bed usage of the next couple of weeks to generate wellness information. 

**Figure 10:**
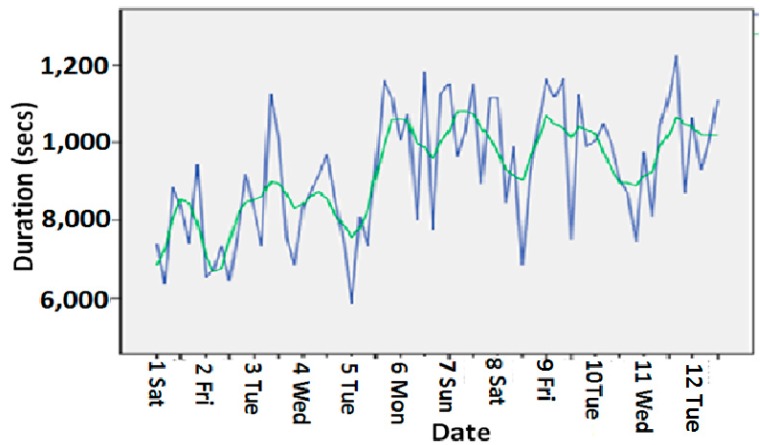
Toilet usage durations and trend.

Figure 10 shows the toilet usage activity durations and the corresponding trend for 11 weeks at a smart home where an inhabitant lives (green color: trend; blue color: actual observations). The trend we had generated from our wellness model was close enough to the actual observations. 

**Figure 11:**
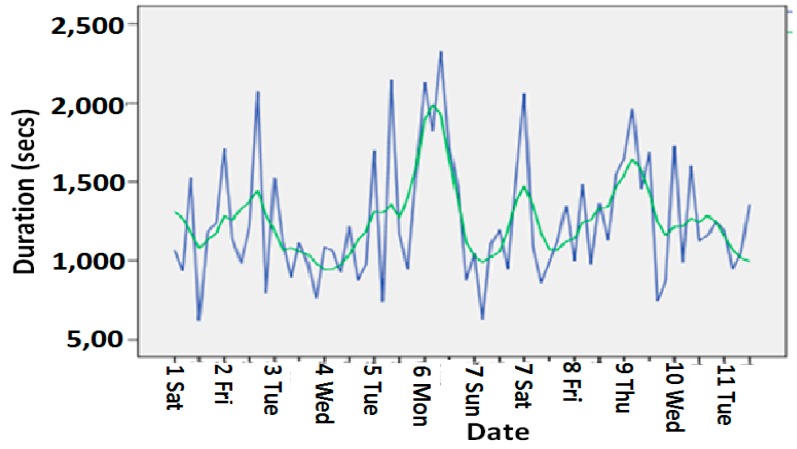
Dining chair usage activity durations trend.

Figure 11 shows the dining chair usage activity durations and the corresponding trend for 11 weeks at an elderly person’s house where he lives alone. The trend we had generated from our wellness model was close enough to the actual observations. 

A snapshot of the ninth week’s (Friday) estimated values based on the recorded eight weeks is given in Table 1. Considering the statistical inference of 95% confidence interval, the residuals in the fitted (predicted) curve is computed by twice the standard deviation. Accordingly, the forecast ranges with maximum and minimum durations are computed according to Equations (1)–(7). 

**Table 1:** Wellness function indices of household appliances and forecast of the ADLs [39].

SUB	Activity	Sensor_ID	β_1_	β_2_	Forecasting for 9th Weeks (Friday)					Actual-Duration (s)	Status
					Max-Time (s)	Min-Time (s)	α	δ	Γ		
#1	Sleeping	Bed	0.715	0.829	28,456	20,405	0.120	0.170	0.650	22,505	Regular
	Dining	Chair		0.865	1208	845	0.020	0.110	0.210	1150	Regular
	Toilet	Toilet		0.785	1444	1028	0.140	0.0900	0.350	1353	Regular
	Relax	Couch		0.889	1457	840	0.150	0.130	0.540	1104	Regular
	Watching TV	TV		0.915	2806	2205	0.030	0.140	0.100	2608	Regular
#2	Sleeping	Bed	0.829	0.727	30,258	23,450	0.200	0.180	0.480	27,268	Regular
	Dining	Chair		0.504	14,545	10,425	0.140	0.170	0.300	15,580	Irregular
	Toilet	Toilet		0.576	1838	1426	0.160	0.130	0.200	1718	Regular
	Relax	Couch		0.614	2018	1487	0.150	0.040	0.250	1645	Regular
	Watching TV	TV		0.813	3804	2807	0.020	0.050	0.060	3045	Regular
#3	Sleeping	Bed	0.604	0.816	27,545	21,408	0.030	0.560	0.605	26,258	Regular
	Dining	Chair		0.713	16,250	12,350	0.400	0.600	0.010	15,145	Regular
	Toilet	Toilet		0.883	1628	1245	0.300	0.450	0.500	1465	Regular
	Relax	Couch		0.615	1845	1423	0.205	0.300	0.150	1628	Regular
	Watching TV	TV		0.715	4055	3605	0.100	0.650	0.750	3810	Regular
#4	Sleeping	Bed	0.758	0.445	28,235	22,035	0.205	0.600	0.700	29,701	Irregular
	Dining	Chair		0.914	1340	950	0.400	0.250	0.010	1205	Regular
	Toilet	Toilet		0.818	1123	885	0.100	0.200	0.650	1060	Regular
	Relax	Couch		0.756	1630	1245	0.300	0.200	0.250	1420	Regular
	Watching TV	TV		0.828	4838	4210	0.040	0.100	0.200	4506	Regular

It was observed that two instances of irregularity at different subject houses were rightly predicted. These were related to the over-usage of the appliances. In reality, the subject was using a chair for a longer time because he was sitting and talking with a guest on that day. In another instance, the duration of bed use shows an over-usage because it was occupied by the elderly person for a long duration as he was unwell. The forecasting procedure has indicated the active durations of the bed and chair were outside the forecast ranges. Accordingly, the behavioral detection process set the status of the corresponding activities as irregular.

## 4. Extension to Smart Building

**Figure 12:**
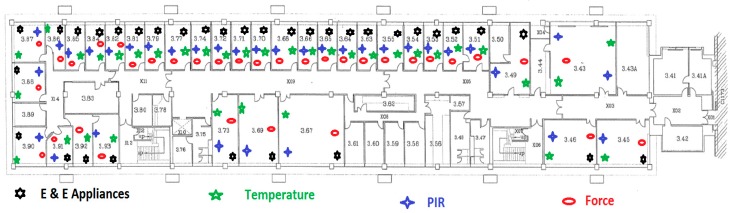
Building layout representing sensors’ placement.

After successful implementation of the smart home, we aim to design and discover the issues related to the smart home monitoring system for building apartment where many people live individually. With the existing methodology of the smart home, we installed the sensing system in a big building apartment, although presently we are covering just one floor that has 35 rooms. The heterogeneous sensing systems scattered across the building with layout is shown in Figure 12. 

**Figure 13:**
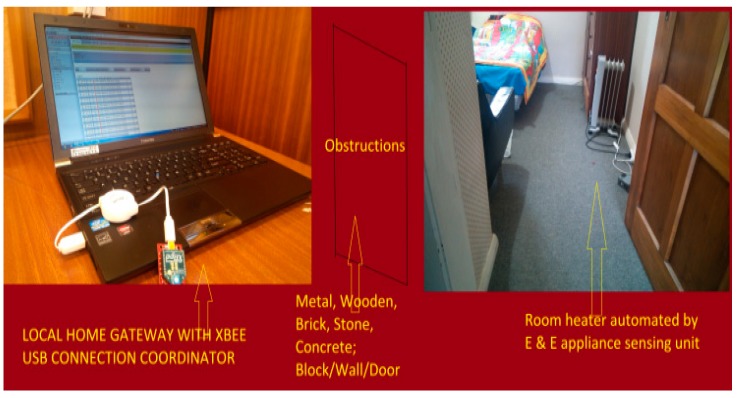
Monitoring system with various types of obstacles.

**Figure 14:**
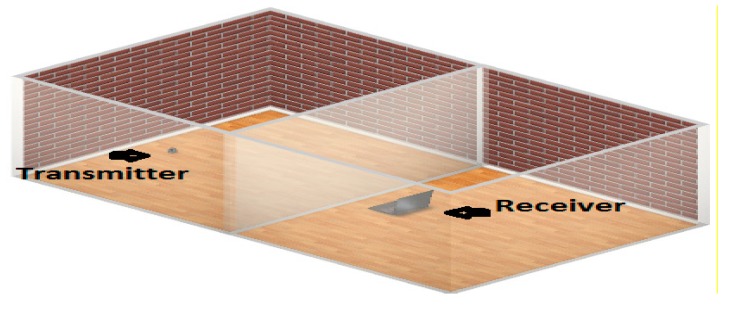
Experimental setup to evaluate the attenuation caused by different building materials in the smart building environment.

Figure 13 presents the overview of building monitoring system setup. The home gateway system that is connected to the coordinator receives the data from heterogeneous sensors scattered in the home environment under various obstructions. Figure 14 shows the smart building experimental setup to evaluate the attenuation loss effect on WSNs. 

The optimum performance in service is only possible with the best reliability. So, for the best reliability user data should be transmitted, received and analyzed within acceptable and defined time duration, in near real time with best precision value and least error.

In WSN-based smart building, explaining the reliability with the system performance perspective is not straightforward, because in the radio communication we find a number of variables that have a negative impact on reliability. The most common explanation of reliability is data reliability; data transmission, reception, measured error and delay. The data transmission and reception are the function of the communication medium between sensor nodes. The quality of this radio communication link enhances the chances of reliable data delivery. Although the design consideration of the sensor node also decides the performance, they are the sources of data, and if data from the source is corrupted then the system cannot achieve the reasonable accuracy. Most of the time, even with good RF link quality and best sensor node design consideration we get less accuracy. The reason of this is improper routing selection and obstruction in the home environment. The urban environment is full of obstacles, so it causes loss of data. Improper routing and topology increase this data loss more, such as when an end device node does not find the nearby router node and that data would be lost. The signal transmission takes place with the speed of light, so the delay is very small or negligible, although we need to consider this latency because in smart home designing we are focusing on real-time intelligence. The combined delay at the sink will be good enough to affect the near real-time streaming.

We can better understand this by the packet reliability terms of ZigBee based WSNs. Packet delivery ratio (PDR), packet success rate (PSR), packet loss rate (PLR), packet error rate (PER), received signal strength, Signal to noise ratio and received packet delay are some of the parameters that define system reliability and performance. In order to evaluate these parameters in IEEE 802.15.4 ZigBee based wireless sensor and networks, the smart building setup is developed and implemented in real-time to get control and monitoring applications without any time delay. The results show that the distance, deployment environment and positioning of sensor nodes are essential parameters that decide the reliability of wireless sensor and networks.

To understand these results, we have to formulate and understand the packet reliability parameters.

Packet delivery parameters: 

The packet sent from the source node must be received at sink node without any distortion and manipulation that affect the accuracy. This is usually determined by the number of messages sent from the source and received at the destination. PDR, PSR, PER, and PLR are closely linked to the packet reliability and system performance, and they represent the packet accuracy at a different level.

- PDR: Packet delivery ratio is the number of packets received at coordinator to number of packets sent from the transmitter; it is represented in percentage.
  (8) $$\text{PDR(\%) }=\frac{{\text{ N}}_{\text{r}}}{{\text{N}}_{\text{s}}}\times 100$$
  ${\text{N}}_{\text{r}}\;$= Total number of packet received by a coordinator from an end device.
  ${\text{N}}_{\text{s}}\;$= Total number of the packet sent by the end device.

The higher the PDR value, the better the system performance and packet reliability.

- PSR: Packet success rate is the number of packets successfully received without any error to number of packets received at coordinator; it is represented in percentage.
  (9) $$\text{PSR(\%)}=\frac{{\text{ N}}_{\text{we}}}{{\text{N}}_{\text{r}}}\times 100$$
  ${\text{N}}_{\text{r}}\;$ = Total number of packet received by a coordinator from an end device.
  ${\text{N}}_{\text{we}}\;$ = Total number of packet received by coordinator without error from an end device.

The higher the PSR value, the better the system performance and packet reliability.

- PER: Packet error rate is the function of number of packets successfully received and the number of packets received at coordinator; it is represented in percentage.
  (10) $$\text{PER(\%)}=\frac{{\text{ N}}_{\text{r}}\;-{\text{ N}}_{\text{we}}}{{\text{N}}_{\text{r}}}\times 100$$
  ${\text{N}}_{\text{r}}\;$ = Total number of packet received by a coordinator from an end device.
  ${\text{N}}_{\text{we}}\;$ = Total number of packet received by coordinator without error from an end device.

The lower the PER value, the better the system performance and packet reliability.

- PLR: Packet loss rate is the function of number of packets received at coordinator and number of the packet sent from the transmitter; it is represented in percentage.
  (11) $$\text{PLR(\%)}=\frac{{\text{ N}}_{\text{s}}\;-{\text{ N}}_{\text{r}}}{{\text{N}}_{\text{s}}}\times 100$$
  ${\text{N}}_{\text{r}}\;$= Total number of packet received by a coordinator from an end device.
  ${\text{N}}_{\text{s}}\;$= Total number of the packet sent by the end device.

The lower PLR value offers the better system performance and packet reliability.

We recognized that other than the distance the location of the end-device in the urban environment affects a lot the performance of the system. The quality of the link is highly degraded by urban environmental obstacles such as door, wall, chair *etc*., followed by ISM band interference and traffic load. 

One of the key issues that affect the reliability and performance of WSN-based smart home is intra-network interference that affects packet delivery. Electromagnetic interference is the disruption that upsets desired node signal processing through the electromagnetic radiation emitted from an external source. ZigBee based sensor nodes operate in the 2.4 GHz ISM spectrum. This intra-network interference becomes more critical in unregulated free ISM band of the frequency spectrum. The disturbance sources in this frequency band include:

- Bluetooth (IEEE 802.15.1)
- Wireless USB version 2 (IEEE 802.15.3)
- Wi-Fi (IEEE 802.11)
- Microwave ovens
- Other sources, like some cordless phones and RF motion detectors [43].

We used MetaGeek Spectrum Analysis Wi-Spy DBx & Chanalyzer 5 device as a frequency spectrum analyzer, and configured it for a particular application requirement [43,44]. We are using density and waterfall graphs to model the interference and loss caused by other RF device in ISM band; these graphs represent the RSSI values at different ZigBee channels. 

The density view plot represents the signal activities in the selected spectrum to recognize the devices; it shows the signal intensity and how often these devices are transmitting. The height of the graph shows the amplitude of the signal, and the color in the plot represents how often the signals are occurring. The more concentrated the color, the more often the frequency is in use; this is called utilization. A blue spike or profile shows a short signal, like a clap. A red spike or shape shows a long, unbroken signal, like an air horn. Colors and their significance are as follows: blue—less than 10% utilization; green—20% utilization; yellow—40% utilization; red—over 50% utilization.

The waterfall view graphs represent amplitude over time for all frequencies in the selected band; much like a seismometer graphs earthquakes. This view is useful for watching the spectrum over time. Unlike the density view, which uses color by utilization, the intensity of the color in the waterfall view shows amplitude. Blue indicates low-amplitude signals while red indicates high-amplitude signals.

**Figure 15:**
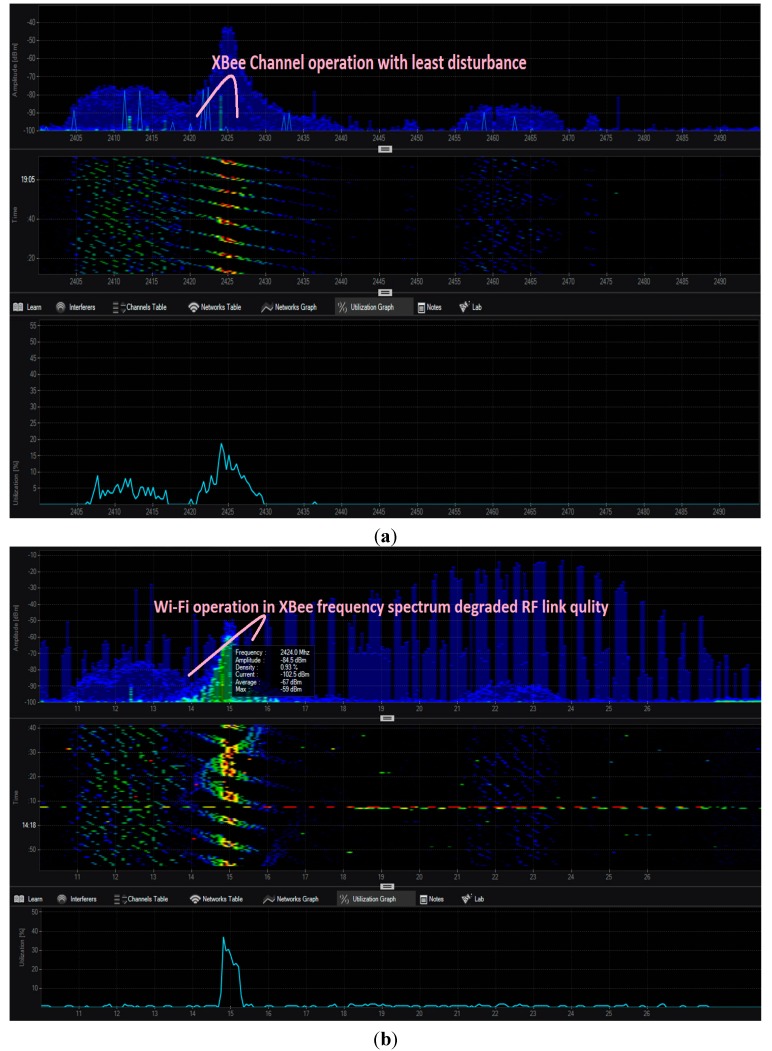

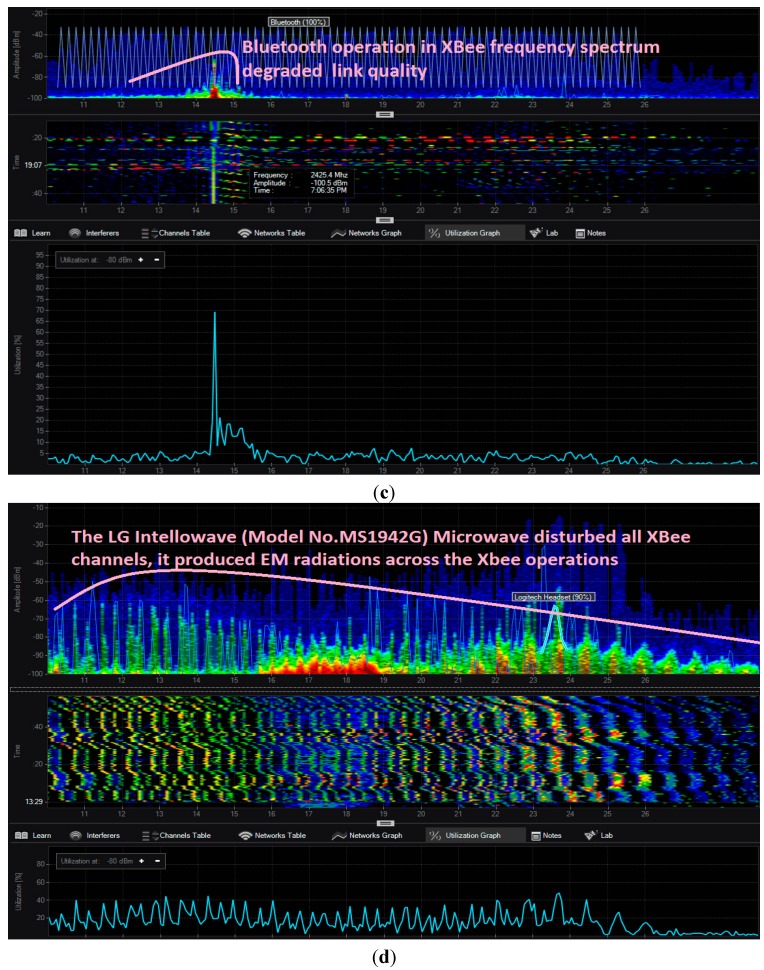
(**a**) shows the that the XBee smart building system is operating at frequency 2.425 MHz; (**b**) shows the Bluetooth functioning over the same frequency 2.425 MHz, which degraded the XBee RF link quality; (**c**) shows the Wi-Fi operation that affected the XBee operation badly; (**d**) Microwave oven distributed all ZigBee channels, and the microwave signal are dissipated across the whole ZigBee spectrum.

Figure 15 shows the interference caused by other wireless technologies to smart home monitoring ZigBee channel. Our ZigBee based WSNs are functioning at channel 2.425 GHz. The sensor nodes are deployed in Mesh topology into a smart building with spacing between nodes is maximum up to 7 m and the Wi-Fi, Bluetooth and microwave sources are placed at 2 m distance from receiver (coordinator). Figure 15a shows the density and waterfall view of XBee smart building system that is operating at frequency 2.425 MHz under least interference condition. Figure 15b shows the Bluetooth functioning over the same frequency 2.425 MHz, which degraded the XBee RF link quality. Figure 15c shows the Wi-Fi operation that affected the XBee operation badly. Figure 15d shows that microwave oven signals are dissipated across the whole ZigBee spectrum. We can better understand this interference effect by the packet reliability parameters shown in Table 2. The packet reliability metrics are most affected by the microwave oven followed by Wi-Fi, and then Bluetooth. 

**Table 2:** Packet reliability parameters affected by different types of ISM band interference sources.

Title	PDR %	PLR %	PSR %	PER %
(a) Least Interference	100	0	99.56	0.44
(b) Bluetooth Interference	99.50	0.50	99.31	0.69
(c) Wi-Fi Interference	97.85	2.15	99.05	0.95
(d) Microwave oven Interference	90	10	96	4

In the smart home monitoring system, most of the time the radio signal does not find the line of sight communication, and it has to travel through multiple obstacles to reach the receiver end. In real conditions, a radio signal usually comes across different kinds of objects in its transmission path and suffers from attenuation, relying on the absorption characteristics of the obstructions. These objects are of different types—such as mobile and static—and absorb the desired RF signal power and attenuate it. In the same way to the free space propagation loss, higher frequencies cause greater losses by attenuation [36,45]. 

The experimental setup arranged for this was a realistic, smart building environment as already represented in Figure 13 and Figure 14 above. In the building environment, we usually find different architectures and various obstructions such as chairs, tables, and other household equipment, but we chose an empty room as well as a hall where we did not find any household stuffs to get a precise measurement of the attenuation caused by the material. Although in the realistic scenario there are different objects and household equipment, these objects add extra attenuation and multipath propagation that affects the measurement. This setup is made to get the fully controlled environment to examine the attenuation effect on system performance. The transmitter and receiver are separated by a particular type of material and the distance to evaluate attenuation loss was defined. 

Initially, with a line of sight path, the RSSI values were recorded and compared with an individual type of obstruction. The attenuation loss by different material types is presented in Figue 16. While the plywood wall does not cause significant attenuation loss, the steel panels used as interior wall in the building causes major attenuation loss. 

**Figure 16:**
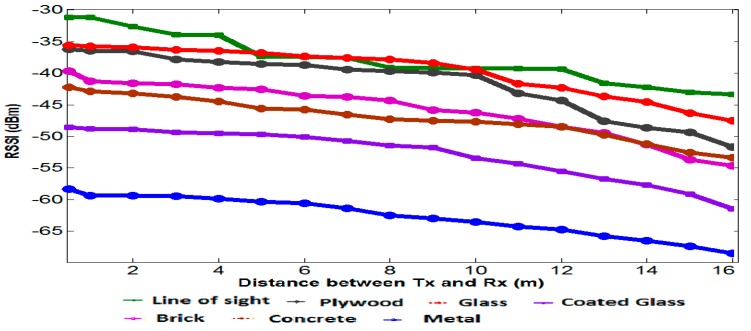
Attenuation loss caused by different materials in the smart building, measured between transmitter and receiver.

Human bodies are made of about 70% water; they attenuate RF signals. The attenuation caused by the human body in ISM band is about 3 dB and in the 5 GHz band it is about 5 dB [36,45].

**Figure 17:**
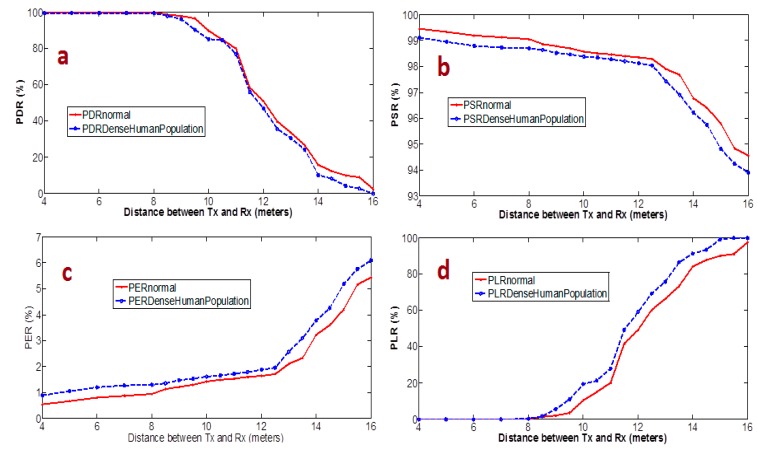
Attenuation loss caused by two different scenarios; one is regular, and other is in a densely populated smart building, measured between transmitter and receiver.

So, to evaluate how a densely populated area affects the system performance, we considered two scenarios, the first was the regular days at home and the second during a party with a group of people celebrating. As shown in Figure 17, the packet reliability in the regular population was slightly better than densely-populated party condition. 

From Figure 17, we can easily identify that there is a marginal improvement in system performance and packet reliability but even this matters. In smart home applications, each and every packet has importance, so even this marginal improvement plays a significant role during any emergency assistance support request.

## 5. Interference and Attenuation Issues and Mitigation 

Digi XBee ZigBee RF device is based on the CSMA-CA but even with that it is not able to mitigate the interference sufficiently. To investigate the characteristics of interference affecting smart building network, we have set up a controlled noise environment. The controlled noise of different noise levels was applied, and the effect at the receiver end was measured [46].

**Figure 18:**
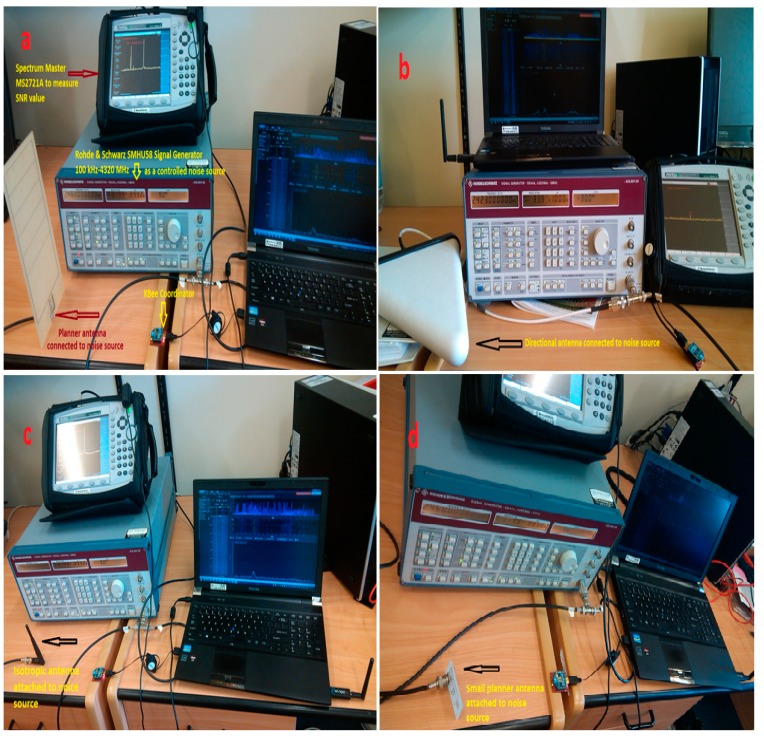
Controlled noise setup to investigate the interference characteristics of the smart building environment; (**a**) External noise generated by planner antenna; (**b**) External noise generated by directional antenna; (**c**) External noise generated by isotropic antenna and (**d**) External noise generated by small planner antenna.

The center frequency of smart building network operation is 2425 MHz. In the smart building environment usually we find the microwave oven, Wi-Fi, Bluetooth and Ham Radio, which operate under ISM spectrum. As shown in Figure 18, to produce controlled noise, we used ROHDE & SCHWARZ 835.8011.58 with different types of antennas, and spectrum analyzer Anritsu MS2127A were used for interference measurement. We did not consider antenna orientation; we only considered the noise source range and frequency band.

**Figure 19:**
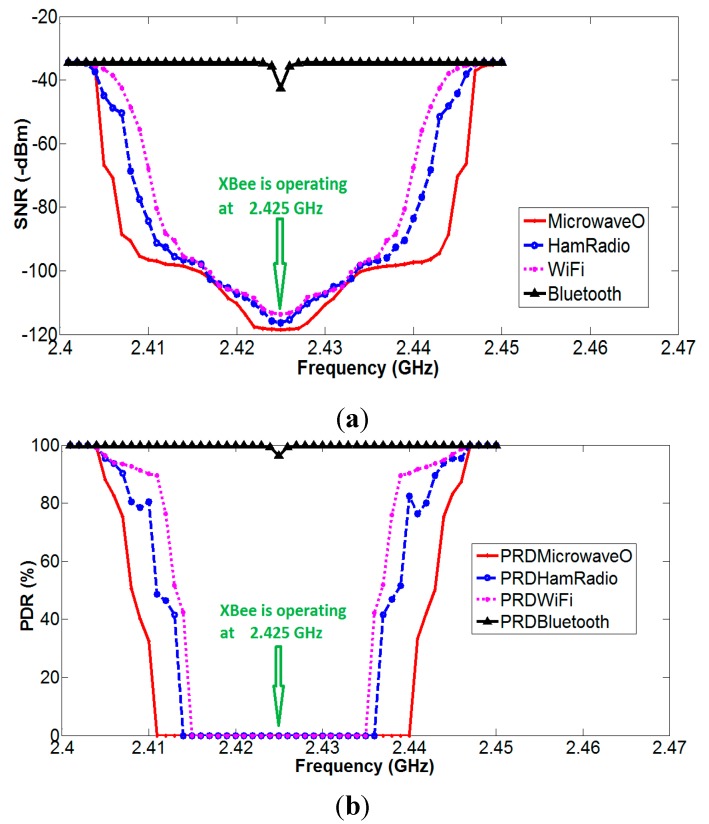
(**a**) SNR *vs.* operating frequency of different RF sources in ISM band; and (**b**) PDR *vs.* operating frequency of different RF sources in ISM band to investigate effect of interferences on ZigBee smart building network.

In obtaining Figure 19, the microwave signal of amplitude 60 dBm occupying the ISM band frequency was applied. Initially, this microwave signal was applied at 2400 MHz, and the corresponding SNR value for ZigBee network was measured, but as the microwave signal operating frequency was moving close to ZigBee network operating frequency, the SNR value was degrading. When it reached the 2425 ± 14 MHz, it had disturbed the whole signal, as we can see by Figure 19, and packet reception became zero. With a similar approach for Wi-Fi, Ham Radio, and Bluetooth, the disturbance regions were identified at 2425 ± 11 MHz and 2425 ± 12 MHz, respectively, whereas Bluetooth did not affect SNR significantly.

One of the best ways is to check the noise level in the ISM band and identify the frequency where the interference is least each time before smart system installation, and select that particular channel for smart building operation. The channel frequency selection is made possible by XBee boot settings.

### Attenuation Mitigation

In the household environment, people usually place different types of material here and there regardless of sensor nodes. When the household stuff is placed near to sensor nodes, it becomes an obstruction for wireless communication. So, the placement of sensor nodes should be done in such a way where it gets least affected by household stuff, and the inhabitants should be made aware to avoid the placement of household stuff near sensor nodes. Changing the building material to reduce the attenuation is not an option for the researcher, but the appropriate placement of sensor nodes in the household environment can reduce the attenuation loss to a significant extent.

## 6. Conclusions

Wireless Sensor Networks and Internet of Things-based smart home is becoming an important ambient-assisted living environment for individuals, where necessary care can be provided at the time of need, and wellness can be measured and predicted. An old home built in 1938 had been converted into a smart home with the help of sensing technology and was in operation since May 2013. The expertise and knowledge of smart home have been explored to extend it to an intelligent building. ISM band interference and attenuation issues have been considered to observe the effectiveness of wireless communication and placement of wireless nodes. More results will be reported in our future works. 
